# Protective effects of Olyset® Net on *Plasmodium falciparum* infection after three years of distribution in western Kenya

**DOI:** 10.1186/s12936-020-03444-w

**Published:** 2020-10-19

**Authors:** Noriko Tamari, Noboru Minakawa, George O. Sonye, Beatrice Awuor, James O. Kongere, Muneaki Hashimoto, Masatoshi Kataoka, Stephen Munga

**Affiliations:** 1 Institute of Tropical Medicine, Nagasaki University, 1-12-4 Sakamoto, Nagasaki, Nagasaki, 852-8523 Japan; 2 College of Public Health, The University of Arizona, 1295 N Martin Ave, Tucson, AZ 85724 USA; 3 Ability To Solve By Knowledge Project, Mbita, Homa Bay, Kenya; 4 Centre for Research in Tropical Medicine and Community Development, Nairobi, Kenya; 5 National Institute of Advanced Industrial Science and Technology (AIST), Health Research Institute, Kagawa, Japan; 6 Centre for Global Health Research, Kenya Medical Research Institute, Kisumu, Kenya

**Keywords:** Llins, Olyset®, DawaPlus® 2.0, *Plasmodium*, Malaria, Repellent effects, Permethrin, Deltamethrin

## Abstract

**Background:**

Several types of insecticides, treating technologies and materials are available for long-lasting insecticide-treated nets (LLINs). The variations may result in different efficacies against mosquitoes and correspondingly infection risks for the *Plasmodium falciparum* malaria parasite. This cross-sectional study investigated whether infection risk varied among children who slept under different LLIN brands in rural villages of western Kenya.

**Methods:**

Children sleeping under various types of LLINs were tested for *P. falciparum* infection using a diagnostic polymerase chain reaction (PCR) assay. Data were collected for other potential factors associated with infection risk: sleeping location (with bed/without bed), number of persons sharing the same net, dwelling wall material, gap of eaves (open/close), proportional hole index, socio-economic status, and density of indoor resting anophelines. Bed-net efficacy against the *Anopheles gambiae* susceptible strain was estimated using the WHO cone test and the tunnel test. The residual insecticide content on nets was measured.

**Results:**

Seven LLIN brands were identified, and deltamethrin-based DawaPlus® 2.0 was the most popular (48%) followed by permethrin-based Olyset® Net (28%). The former LLIN was distributed in the area about six months before the present study was conducted, and the latter net was distributed at least three years before. Of 254 children analysed, *P. falciparum* PCR-positive prevalence was 58% for DawaPlus® 2.0 users and 38% for Olyset® users. The multiple regression analysis revealed that the difference was statistically significant (adjusted OR: 0.67, 95% credible interval: 0.45–0.97), whereas the confounders were not statistically important. Among randomly selected net samples, all DawaPlus® 2.0 (n = 20) and 95% of Olyset® (n = 19) passed either the cone test or the tunnel test.

**Conclusions:**

Olyset® was more effective in reducing infection risk compared with DawaPlus® 2.0. Although the data from the present study were too limited to explain the mechanism clearly, the results suggest that the characteristics of the former brand are more suitable for the conditions, such as vector species composition, of the study area.

## Background

Long-lasting insecticide-treated nets (LLINs) are effective in reducing malaria morbidity and mortality [1–3] and are widely accepted as an important tool to control malaria parasite infection [4]. As of 2019, the World Health Organization (WHO) listed 20 LLIN brands for procurement by international agencies and countries [5]. The pyrethroid insecticides used for these LLINs are deltamethrin, alpha-cypermethrin, and permethrin [5]. The insecticides act differently against anopheline mosquitoes; for instance, a laboratory study showed that fabric treated with deltamethrin has better killing effects than one with permethrin; however, the latter shows stronger repellent effects [6–8]. Although both types of pyrethroids reduce human contact with anophelines by killing and repelling, the question remains which mode of action or insecticide is better for reducing infection risk. To counter anophelines resistant to pyrethroids and dichlorodiphenyltrichloroethane (DDT), five LLIN brands in the list are treated with piperonyl butoxide (PBO) in addition to the pyrethroids. A randomized control trial showed that LLINs treated with PBO and permethrin are more effective in reducing *Plasmodium falciparum* parasite infection than nets with insecticide only [9].

The difference in insecticide treating technology and LLIN materials may also affect their performance. One treating technology is called incorporation technology; specifically, an insecticide is incorporated into polyethylene-based fibres of the net [10], such as the Olyset® Net (Sumitomo Chemical, Tokyo, Japan). The insecticide migrates from the inside of the fibres to the surface so that the amount of the active content is maintained for several years. Coating is a second technology, in which the polyester-based multifilaments are coated with insecticide using a resin-based polymer [11] that serves as a reservoir for replacement of insecticide lost from the surface. Polyethylene and polyester are the two major materials used for LLINs. The difference in treatment technology and netting material may alter the durability of nets and the availability of insecticides on the fibre surfaces and, consequently, affect infection risks [12, 13].

A meta-analysis showed that among three different insecticides the differences in effectiveness on infection risk were not statistically significant [3]. A large population-based cross-sectional study using data from 21 countries across sub-Saharan Africa also found little variability on infection of children by LLIN brands [14]. However, when data were examined separately for each surveyed population, the effects of LLINs varied among LLIN brands for some populations [14, 15]. Local environmental conditions likely vary across large geographical malarial areas and may influence the performance of LLINs. Local vector species compositions and their insecticide resistance status can also contribute to the variability of LLIN performance [16–20].

Since a variety of LLIN brands are available, and the environmental conditions of target areas vary, an LLIN brand that is effective for a certain area might be less effective elsewhere, and, therefore, the selection of an appropriate LLIN brand may become important. More comparative studies of various LLIN brands in real-field conditions are needed to produce information to select the most suitable LLIN brand for a target area. This study investigated whether *P. falciparum* parasite infection risk varies among children who slept under different LLIN brands, along with various conditions in rural villages in a malaria hyperendemic area of western Kenya.

## Methods

### Study area and target population

**Fig. 1:**
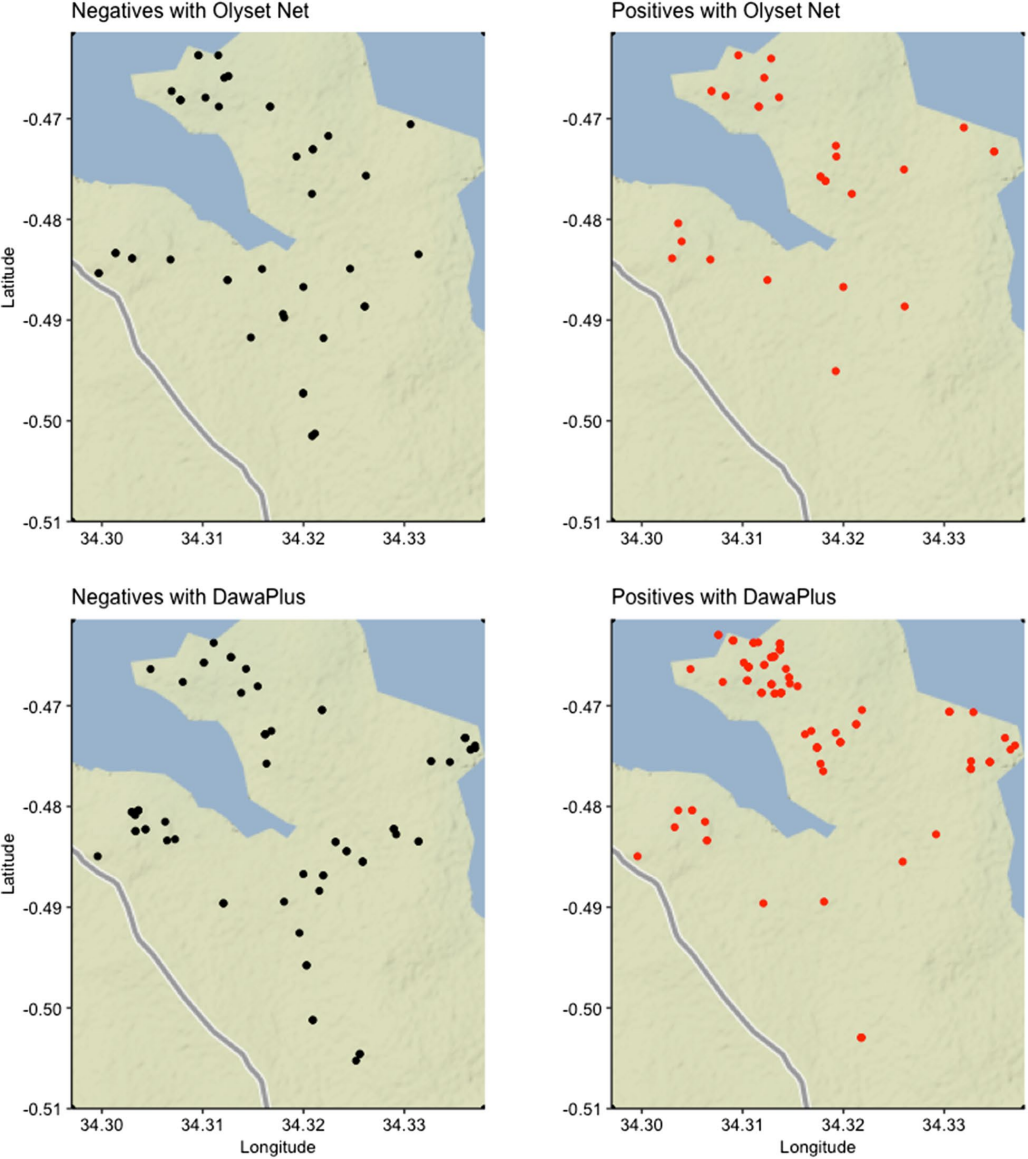
Distributions of children who used Olyset® Net and DawaPlus® 2.0, and their PCR-*Plasmodium falciparum* infection status

The study area was located on the western part of Gembe East Sub-location in Homabay County, Kenya (approximate area: 12 sq km; approximate midpoint: 0° 28′ 24.06″ S, 34° 19′ 16.82″ E) (Fig. 1). For scheduling and logistics, the area was divided into 12 sub-areas based on community centres, villages and political boundary. The rainfall pattern was binomial, with a long rainy season from March through May and a short rainy season from November to December. A typical household compound consists of multiple mud house structures with corrugated iron roofs that have open eaves [21–23]. Principal economic activities are small-scale fishing, farming and livestock [21–23].

In December 2017, the population of the area was estimated to be 3769 through a household survey; the initial target population was children 15 years old and younger. Of 2006 children registered, 1094 were excluded because the ceilings of their sleeping houses were screened with a LLIN material in a previous project, which may affect the risk of infection [24]. The number of remaining children was 912.

### Informed consent

Prior to the survey, the field workers visited households that consisted of at least one eligible child and explained the details of the survey, including the goals, risks and benefits. Once written consent was obtained from household heads or caretakers of the children, they were informed of the dates and time of the survey. At the time of visit, geographical coordinates of house structures where the children slept were recorded using a handheld global positioning system (GPS) (Garmin, Olathe, KS, USA).

### Field data collection

Because the data on *P. falciparum* parasite infection of children were provided from the field evaluation study for developing a rapid diagnostic device [25], this study followed the schedule of the evaluation study. Accordingly, the cross-sectional survey was conducted separately for 6 of the 12 sub-areas in December 2017 and for the remaining sub-areas in February 2018.

The field workers visited the households between 05:00 and 06:30, and directly observed bed net use and sleeping locations of the target children, because these variables may affect infection risk [21, 23, 26–29]. Sleeping location was categorized as ‘with bed’ or ‘without bed’. Cases of sleeping on the floor, sofa or mattress without a bed frame were considered as ‘without bed’. The field workers also recorded the number of persons sharing the same net with each target child. The children were divided to two groups based on the number of persons sharing a net, ‘children sharing with none or one person’ and ‘those sharing with two or more persons’. This is based on the WHO recommendation of ‘one net for every two persons’, and the protective effect of LLIN that is expected if the recommendation is followed [30].

Immediately after the direct observation, indoor-resting female anophelines were sampled in the rooms where children slept, using the pyrethrum spray catch method [31]. The area (sq m) of each room was estimated, measuring with a tape measure, and the density of female anophelines was calculated as the number of anophelines divided by the area of sleeping room. All mosquitoes were collected before the local government started the indoor residual spray programme prior to the long rainy season of 2018.

Since modern house structures may reduce the infection risk, the field workers recorded wall materials (mud/others) and the presence/absence of eave openings [29, 32]. Cement, brick and iron walls were included in ‘others’ for wall material, because those houses were uncommon in this study area. In addition, household heads were asked if they possessed various consumer goods, materials of household construction, toilet/water access, and livestock, for constructing a socio-economic status (SES) index using multiple correspondence analysis [33–35].

During the household survey, a finger-prick blood was sampled from children after their axillary temperature was measured. The blood sample was tested for *P. falciparum* infection with a rapid diagnostic test (RDT)(Paracheck® Pf-Rapid Test Malaria Device, ver.3, Orchid Biomedical Systems, Verna, Goa, India). A part of the blood sample was used for measuring haemoglobin concentration with a portable haemoglobin photometer (HemoCue AB, Angelholm, Sweden). Artemether-lumefantrine was given to each child following diagnosis by a clinician and the guideline issued by WHO [36]. Children with haemoglobin concentration below 11.0 g/dL were given iron supplementation. In the laboratory, the blood samples were examined to detect *P. falciparum* using a diagnostic polymerase chain reaction (PCR) assay [37].

At the end of the survey, the bed nets that the children used were collected and new nets provided. Brand names of the bed nets were identified, and the proportional hole index (PHI) was estimated for each net following the WHO guideline [38]. PHI of the roof and side was calculated separately because more mosquitoes enter the net from the roof holes compared to the side holes [39, 40].

Data were collected on paper forms. Data entry was performed by two persons and independently verified. When discrepancies or missing data were found, the field workers revisited the households to confirm the data.

### Insecticidal activity of LLIN

Following the WHO guideline for the bioassay test and chemical analysis [41], 5 pieces of nettings (4 pieces from side panels and one piece from the roof panel, 25 cm × 25 cm) were cut from each of 20 randomly selected nets for each LLIN brand. In the laboratory the biological efficacy of the nets was evaluated in the WHO cone bioassay test using the susceptible *Anopheles gambiae s.s.* Kisumu strain. Batches of five, non-blood-fed, 2–5-days-old females were exposed to each piece of netting in WHO cones for 3 min, and then the mosquitoes were held for 24 h with a sugar solution. A total of 100 females were exposed to each netting (5 mosquitoes × 5 cones × 4 pieces). Knockdown rate and mortality rate were observed at 60 min and 24 h after exposure, respectively. The WHO tunnel test using the *An. gambiae* Kisumu strain was conducted for netting that did not pass the cone test to determine the blood-feeding inhibition rate and 24-h mortality. Residual insecticide content of Olyset® and DawaPlus® 2.0 was analysed based on the CIPAC method 331/LN/M and 333/LN/(M)/3, respectively.

### Power calculation

A study in the Democratic Republic of Congo had 32% of *P. falciparum* PCR-positive prevalence (pfPR) for children under 5 years of age who slept under deltamethrin-based nets, while the pfPR was 42% for those slept under permethrin-based nets [15]. With a Type I error of 5% and a sample size of 254, the power to detect the difference between deltamethrin-users and permethrin-users was 64%. Since the epidemiological data of the children were provided from the diagnostic device field evaluation study [42], the present study proceeded despite the relatively low detection power.

### Ethical consideration

This study was approved by the Ethics Committees of the Kenya Medical Research Institute (SSC No. 3168), and the Ethics Committees of the National Institute of Advanced Industrial Science and Technology (No. 2017-156).

### Data analyses

Association of 10 covariates with bed net brands was assessed using the Chi-square test for binary and categorical variables, and the Wilcoxon rank-sum test or t-test for continuous variables [29]. The covariates were number of persons sharing a net, age, gender, sleeping location, PHI of the roof, PHI of the sides, SES, density of female anophelines in the room, material of wall, and eave gap. Simple logistic regression examined the impacts of bed net type and the 10 variables on pfPR of children.

A multiple logistic regression model of parasitaemia examined bed net type and the 10 confounding factors. Generalized variance inflation factor (GVIF) was used to assess collinearity among the covariates [43]. Since the survey was conducted on multiple dates during the two separate periods, the dates were considered as a random effect. When multiple target children shared the same net and sleeping room, those nets and rooms were also considered as potential random effects. Further sub-area was included as a potential random effect. Model selection based on the deviance information criteria (DIC) was used for testing whether each random effect was necessary. The variogram was used to assess the presence of spatial dependency in the residual for each analysis. DIC was also used to assess whether the spatial model was better than the non-spatial model. The spatial component was included in the Bayesian model using integrated nested Laplace approximation (R-INLA) with 95% credible intervals [44].

The residual active insecticide content was compared between net brands using a generalized liner mixed model (GLMM) with 95% confidence intervals. The original LLIN from which 5 sub-sample nettings were obtained was considered as a random factor in the model. Knockdown rate and mortality rate were examined using Chi-square test. R (version 3.5.2) was used for all data analyses [45].

## Results

### Target children and LLINs

Of 912 target children, 729 were available at the time of the survey, and 546 slept under a LLIN. The number of LLINs used was 307, and 7 different LLIN brands were identified. DawaPlus® 2.0 (Tana Netting, Dubai, UAE) was the most popular (48%) followed by Olyset® (28%). The rest were Yorkool® LN (4%; Tianjin Yorkool International Trading, Tianjin, China), DuraNet® (4%; Shobikaa Impex, Tamilnadu, India), PermaNet® 2.0 (2%; Vestergaard, Lausanne, Switzerland), Olyset®Plus (1%; Sumitomo Chemical, Tokyo, Japan), Netprotect®(0.3%; Bestnet A/S, Kolding, Denmark), and unidentified nets (12%). Because DawaPlus® 2.0 and Olyset® comprised 76% of all the LLINs, the following analyses focused on these two brands. The Ministry of Health of Kenya distributed DawaPlus® 2.0 through health facilities in the study area in June and July, 2017, and Nagasaki University distributed Olyset® in September and October 2014.

**Fig. 2:**
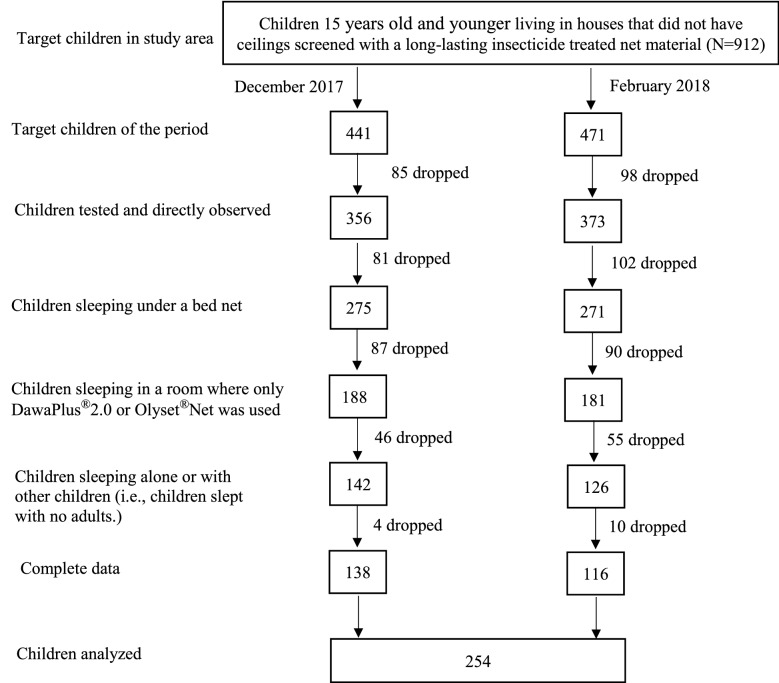
Flowchart showing selection of children

In December 2017, 356 children were directly observed for their sleeping conditions and tested for *P. falciparum* parasite infection (Fig. 2). Of these children, 139 were dropped because they did not use bed nets, or slept under nets other than Olyset® or DawaPlus® 2.0. Of 217 children remaining, 27 children shared the same room with other persons who did not use bed nets or slept under other types of nets, and 48 children shared the same room where at least one adult slept; the exclusion would minimize the influence of presence of a non-net user, other net types and adults. Adults may occupy a large space inside a net, and children might be squeezed towards the net, which may increase the risk of touching it or extending their limbs outside of it. On the other hand, parents tend to lay their small child (particularly a child under 2 years old) between their bodies in the net, which may provide protection. After removing 4 children without complete data, 138 children remained. In the same manner, 355 were dropped from 471 children who were available for the survey, and 116 children remained for the survey in February 2018. In total, a dataset of 254 children was used for the analyses.

## Background information

The mean child age was 8.4 (SD = 3.5) years old, and the difference was not statistically significant between DawaPlus® 2.0 users and Olyset® users (Table 1). The differences in gender ratio and sleeping location were also not statistically significant between the two groups. The PHI on the side of Olyset® was significantly greater than that of DawaPlus® 2.0 while the difference in PHI on the roof was not significant. The proportion of children sharing the same net with two or more persons was significantly higher for DawaPlus® 2.0 users. The proportions of mud wall and open eaves were significantly higher for the rooms where DawaPlus® 2.0 users slept, and the SES of DawaPlus® 2.0 users was significantly lower. A total of 1365 anopheline mosquitoes were collected from 118 rooms where 254 target children slept. Although the mean density of anophelines in the rooms with Olyset® was almost half compared to those with DawaPlus® 2.0, the difference was not statistically significant.

**Table 1:** Association of each explanatory variable with DawaPlus® 2.0 and Olyset®Net

Parameter	DawaPlus® 2.0 n = 180 (70.9)^a^	Olyset®Net n = 74 (29.1)	p-value
Age	8.24 ± 0.26^2^	8.89 ± 0.42	0.19
Density of female anophelines in the room (/m^2^)	1.32 ± 0.16^2^	0.68 ± 0.09	0.09
Gap of eaves			
Close	10 (5.6)^a^	14 (18.9)	< 0.01*
Open	170 (94.4)	60 (81.1)	
Gender			
Female	78 (43.3)^a^	38 (51.4)	0.24
Male	102 (56.7)	36 (48.6)	
Material of wall			
Other than mud	18 (10.0)^a^	15 (20.3)	0.03*
Mud	162 (90.0)	59 (79.7)	
PHI^a^			
On the roof	0 (0–1599)^c^	0 (0–2061)	0.12
On the sides	0 (0–5529)^c^	0 (0–7497)	< 0.01*
No. of persons sharing a net			
Share with none or 1 person	73 (40.6)^a^	47 (63.5)	< 0.01*
Share with 2 persons or more	107 (59.4)	27 (36.5)	
Sleeping location			
Without bed	151 (83.9)^a^	55 (74.3)	0.08
With bed	29 (16.1)	19 (25.7)	
Socioeconomic status	0 ± 0.02^b^	0.15 ± 0.04	0.01*

^*a*^*PHI* Proportional hole index

^a^(%), ^b^mean ± standard error, ^c^Median (range)

*Statistically significant

### Insecticidal activity

The mean residual insecticide content was 14.11 g/kg (SD = 3.31, n = 94) and 0.40 g/kg (SD = 0.29, n = 100) for Olyset® and DawaPlus® 2.0, respectively. When the contents were compared with the original contents (20.0 g/kg for Olyset®, and 2.66 g/kg for DawaPlus® 2.0), the residual rate was 70% and 15% for Olyset® and DawaPlus® 2.0, respectively. The residual rate was significantly higher for Olyset® (OR: 1.73, 95% confidence interval: 1.60–1.89, n = 194). Six Olyset® nettings were removed from the analysis because of measurement errors.

The knockdown rate after 60 min was 98 and 99% for Olyset® and DawaPlus® 2.0, respectively, and the difference was not statistically significant (OR = 0.98, $\chi$ ^2^ = 0.18, P = 0.668, df = 1). The 24-h mortality was 52 and 92% for Olyset® and DawaPlus® 2.0, respectively, a statistically significant difference (OR: 0.56, $\chi$ ^2^ = 129.81, P < 0.001, df = 1). The tunnel test was conducted for two Olyset® and one DawaPlus® 2.0 that did not pass the cone test. While one Olyset® did not pass the tunnel test, the others passed the test. In total, 18 of 19 Olyset® (95%) and all DawaPlus® 2.0 passed either the cone test or the tunnel test. Data from one Olyset® was removed because of measurement errors.

### Plasmodium falciparum infection

The pfPR was 58% for DawaPlus® 2.0 users and 38% for Olyset® users (Fig. 1). The simple logistic regression analysis showed that the 95% credible interval did not contain 0 for the net brands (Additional file 3: Fig. S3 and Table 2). The 95% credible intervals for the other variables contain 0. All analyses included the spatial component because of presence of spatial dependency (Additional file 1: Fig. S1).

**Table 2:** Results from simple and multiple logistic regression that measured the impact of type of bed nets and the confounding factors on PCR-positive prevalence of children at the age of 15 years old and below (N = 254)

Parameter	Negative n = 122 (48.0)^a^	Positive n = 132 (52.0)	Bivariate		Multiple	
			OR	95% Credible interval	OR	95% Credible interval
Type of bed nets						
DawaPlus® 2.0	76 (42.2)^a^	104 (57.8)	–	–	–	–
Olyset®Net	46 (62.2)	28 (37.8)	0.67	[0.48, 0.91]	0.67	[0.45, 0.97]
Age	8.66 ± 0.33^2^	8.23 ± 0.30	0.80	[0.60, 1.07]	0.87	[0.63, 1.19]
Density of female anophelines in the room (/m^2^)	1.01 ± 0.19^2^	1.24 ± 0.14	1.13	[0.84, 1.53]	0.94	[0.61, 1.33]
Gap of eaves						
Close	18 (75.0)^a^	6 (25.0)	–	–	–	–
Open	104 (45.2)	126 (54.8)	1.34	[0.97, 1.88]	1.14	[0.75, 1.72]
Gender						
Female	55 (47.4)^a^	61 (52.6)	–	–	–	–
Male	67 (48.6)	71 (51.4)	1.06	[0.80, 1.42]	0.98	[0.71, 1.34]
Material of wall						
Other than mud	22 (66.7)^a^	11 (33.3)	–	–	–	–
Mud	100 (45.2)	121 (54.8)	1.24	[0.90, 1.74]	1.08	[0.70, 1.70]
PHI						
On the roof	0 (0–1619)^c^	0 (0–2061)	1.40	[0.96, 2.27]	1.50	[0.96, 2.64]
On the sides	0 (0–7497)^c^	0 (0–7497)	1.00	[0.74, 1.35]	0.94	[0.63, 1.39]
No. of persons sharing a net						
Share with none or 1 person	68 (56.7)^a^	52 (43.3)	–	–	–	–
Share with 2 persons or more	54 (40.3)	80 (59.7)	1.22	[0.90, 1.67]	1.14	[0.80, 1.66]
Sleeping location						
Without bed	93 (45.1)^a^	113 (54.9)	–	–	–	–
With bed	29 (60.4)	19 (39.6)	0.89	[0.65, 1.21]	1.00	[0.69, 1.44]
Socioeconomic status	0.12 ± 0.03^2^	− 0.02 ± 0.03	0.75	[0.54, 1.04]	0.91	[0.56, 1.43]

Random effects for household areas and rooms of mosquito collection were included in the simple and multiple logistic regression analyses

^a^(%), ^b^mean ± standard error, ^c^median (range)

The multiple logistic regression analysis confirmed the results from the simple regression analysis (Table 2). When Olyset® was used, the risk of infection became lower, and the 95% credible interval did not contain 0 (Additional file 4: Fig. S4). The GVIFs for the all covariates were less than 2 so they were included in the analysis. Among the potential random factors, date of survey and bed net were dropped from all analyses after model selection using deviance information criteria (DIC). Sub-area and sleeping room remained as random effects. All analyses considered spatial dependency, because the spatial models had slightly better sample variograms (Additional file 2: Fig. S2).

## Discussion

This study demonstrated that the pfPR of children differed between users of Olyset® and DawaPlus® 2.0. The odds ratio indicated that use of Olyset® for a child reduced by 33% the likelihood of being infected (Table 2). The study also found differences in some variables between two groups (Table 1). The households of Olyset® users had significantly higher SES, and a decrease of infection risk is often associated with an increase of SES [46]. Households with low income cannot easily afford extra protection such as insecticide spray and drugs. Moreover, parents of households with high SES tend to have a better health knowledge, which may also lower the risk [47, 48]. It is plausible that households with high SES have better house construction, with glass or screened windows and closed eaves to prevent mosquitoes from entering [49]. While most of the houses in the study area were constructed with a mud wall and corrugated iron roofs that have open eaves, the higher proportion of the rooms with Olyset® had closed eaves and non-mud walls, such as concrete. Although the difference was not statistically significant, the density of anophelines in the rooms of Olyset® users was nearly 50% less than in those of DawaPlus® 2.0 users, suggesting that Olyset® users might have lower infection risks because of higher SES (Additional file 3: Fig. S3, Additional file 4: Fig. S4).

In addition to freely distributed LLINs, households with higher SES might be able to buy extra nets. Although the WHO guideline suggests ‘one net for every two people’ to achieve universal coverage, the goal has not been reached in several areas [50], where one net is often shared by more than two persons. The number of people sharing a net is significantly higher among DawaPlus® 2.0 users compared to Olyset® users. A recent study confirmed that the risk increases with an increase in the number of people sharing one LLIN, because the condition may increase the chance of their body being exposed outside the net [30].

As Olyset® in the study area had been used by residents longer than DawaPlus® 2.0, the condition of the former net was worse. The PHI on the sides of Olyset® was significantly higher. Although the difference was not statistically significant, the Olyset® also had a higher PHI on the roof. As mosquitoes may enter through holes, the infection risk is expected to be higher for children sleeping under the old nets. However, the results from the present study did not agree with this notion. Even though the effects of all these confounding factors, including spatial dependency, were controlled in the multiple regression model, the difference in pfPR between Olyset® users and DawaPlus® 2.0 users still remained.

The difference in pfPR of children sleeping under these LLIN brands may be explained by the differences between the insecticides. Permethrin is incorporated in the fibres of Olyset® while DawaPlus® 2.0 is treated with deltamethrin on the filament surface. A laboratory experiment showed that *An. gambiae* reduced landing attempts on Olyset® and increased frequencies of flight after the first contact with the net, while landing attempts on the net treated with deltamethrin were sustained longer [7, 51]. A possible effect of disengagement behaviour associated with permethrin is loss of the ability to sense host cues, which is also known in other insects, such as *Glossina austeni* [52]. Because of the greater repellent effect of permethrin, Olyset® may reduce the number of anophelines by decreasing the attraction cues of the room [53, 54]. The lower density of anophelines associated with Olyset® found in the present study agrees with this notion although the lower density cannot be explained by the data from the spray catch method only.

The disengagement associated with permethrin suggests decreased interactions between mosquitoes and humans [7, 55]. This deterrence effect should be beneficial for children sleeping with bed nets having holes [54, 56], and multiple children sharing one net [30]. Mosquitoes exposed to permethrin will not seek a blood meal even under these conditions because of a loss of the ability to sense host cues. Similarly, the deterrence will be beneficial for persons who are not under an LLIN [56, 57]. Further, this effect may reduce early-hour biting activity in the room or even outdoor biting activity near a house with an Olyset® [8]. Although loss of the response to host cues may be restored within 24 h [7], as long as a mosquito repeatedly visits the net with permethrin, the effect can be sustained. Permethrin is continuously provided to the surface of the fibre from the inside via osmotic pressure [10]. The present study confirmed that Olyset® used for at least three years had sustained the efficacy.

It is known that mosquito disengagement from Olyset® reduces lethality [7, 51]. The low 24-h mortality of Olyset® found in the bioassay of the present study is also likely due to disengagement, but it is not due to net age because enough insecticide content was maintained on the surface. It has been suggested that the poor killing effect delays the development of resistance against the insecticide [16, 54, 56]. Three main vector species in western Kenya have developed resistance to pyrethroid insecticides; namely, *An. gambiae *sensu stricto (*s.s.*), *Anopheles arabiensi*s and *Anopheles funestus s.s.* Specifically, *An. gambiae s.s.* has developed resistance associated with a point mutation (knockdown resistance mutation: *kdr*) in the voltage-gated sodium channel (L1014S), and the other two species have developed metabolic resistance related to one or more detoxification enzymes, such as cytochrome P450s [16, 17, 58]. Although the field-collected adults of the three species from the present study area show strong resistance to both deltamethrin and permethrin in the susceptibility test using the WHO tube test, permethrin still shows a strong repellent effect against *An. arabiensis* and *An. funestus s.s.*[8]. On the other hand, permethrin has a less repellent effect against *An. gambiae* with *kdr* [6, 8]. As *An. arabiensis* and *An. funestus* dominate the present study area after the disappearance of *An. gambiae* [59], the Olyset® might have become more effective. In contrast, studies in Democratic Republic of Congo as well as Benin demonstrated that use of deltamethrin-based LLINs is associated with lower pfPR than use of permethrin-based LLINs [14, 15]. This conflicting result may be due to great abundance of *An. gambiae* or *Anopheles coluzzi* with *kdr* in these countries [60–63]. Although the present study excluded the potential effects of other LLIN brands in the same room, the study in Democratic Republic of Congo apparently did not exclude them because of a large-scale cross-sectional study.

This study collected data on the types and locations of LLINs. Although residents were not asked where they preferred to hang what kinds of nets, there were some important commonalities. Olyset® were more common around beds, where nets were hung in a semi-permanent fashion. DawaPlus® 2.0 were more common in community living areas, where nets must be hung, taken down and stored to make the space available for other activities during the daytime [26]. The Olyset® fabric is hard and difficult to fold to a compact size for storage possibly making it a preferred type of net in sleeping areas. The DawaPlus® 2.0, on the other hand, are softer and easier to fold on a routine basis which could explain why residents prefer to use it in living rooms and gathering spaces. This practice can explain not only the positive association of Olyset® users with sleeping in a bed, but also their negative association with pfPR. Unremoved Olyset® may strengthen the deterrence effects increasing the chance of mosquito contact with it even though no one is under the net, because human odour remaining on bedding would be still strong enough to attract mosquitoes to the net. Especially, the deterrence effects may reduce mosquito bites of residents outside of the nets in the early morning and evening. The early-hour biting is characteristic of *An. arabiensis* and *An. funestus* in this study area [64]. Moreover, simply using a net in the bed may provide more protective effects compared to sleeping on the floor, because the net spreads well with the bed frame, and its bottom end is tucked in firmly under the mattress [23].

## Limitations

Data from the present study were insufficient for explaining the lower pfPR associated with Olyset® use. Data collection was limited to the low transmission season to match the schedule of the previous study. Since vector abundance and species composition may vary seasonally, the effects of LLINs might change during the high transmission season. As some residents alter their sleeping locations and bed nets, a cross-sectional study will have some limitations. In particular, the factors associated with bed net use might affect the results because the skewed sample size of Olyset® users was less than half of DawaPlus® 2.0 users. More studies under different locations and conditions are needed to confirm the repellent effects of LLINs with permethrin on *P. falciparum* parasite infection in the field.

## Conclusions

The present study showed the possibility that *P. falciparum* parasite infection varies depending on the LLIN brand used. The results from the present study suggest that a selection of LLIN brand requires more care to maximize their effectiveness. The selection will be based on the environmental condition of each target village or geographical area. In particular, vector species composition and their insecticide resistant status should be considered for the selection.

## Supplementary information

**Additional file 1: Fig. S1.** Variograms to examine spatial dependency in the simple regression model for each explanatory variable.

**Additional file 2: Fig. S2. **Variograms to examine spatial dependency in the multiple regression model.

**Additional file 3: Fig. S3.** The 95% credible intervals of each variable from non-spatial and spatial bivariate regression models.

**Additional file 4: Fig. S4.** The 95% credible intervals of each variable from non-spatial and spatial multiple regression models.

## Data Availability

The datasets used and/or analysed during the current study are available from the corresponding author on reasonable request.

## Abbreviations

DDT: Dichlorodiphenyltrichloroethane
DIC: Deviance information criteria
GLMM: Generalized liner mixed model
GPS: Global positioning system
GVIF: Generalized variance inflation factor
IRS: Indoor residual
*kdr*: Knockdown resistance mutation
LLIN: Long-lasting insecticide-treated net
PBO: Piperonyl butoxide
PCR: Polymerase chain reaction
pfPR: *P. falciparum* PCR-positive prevalence
PHI: Proportional hole index
SES: Socioeconomic status
RDT: Rapid diagnostic test
R-INLA: Integrated Nested Laplace Approximation
WHO: World Health Organization
