# Supplementary figures and images for: Protective effects of Olyset® Net on Plasmodium falciparum infection after three years of distribution in western Kenya
Source: Malar J. 2020 Oct 19;19:373. doi: 10.1186/s12936-020-03444-w (PMC7574443; doi:10.1186/s12936-020-03444-w)

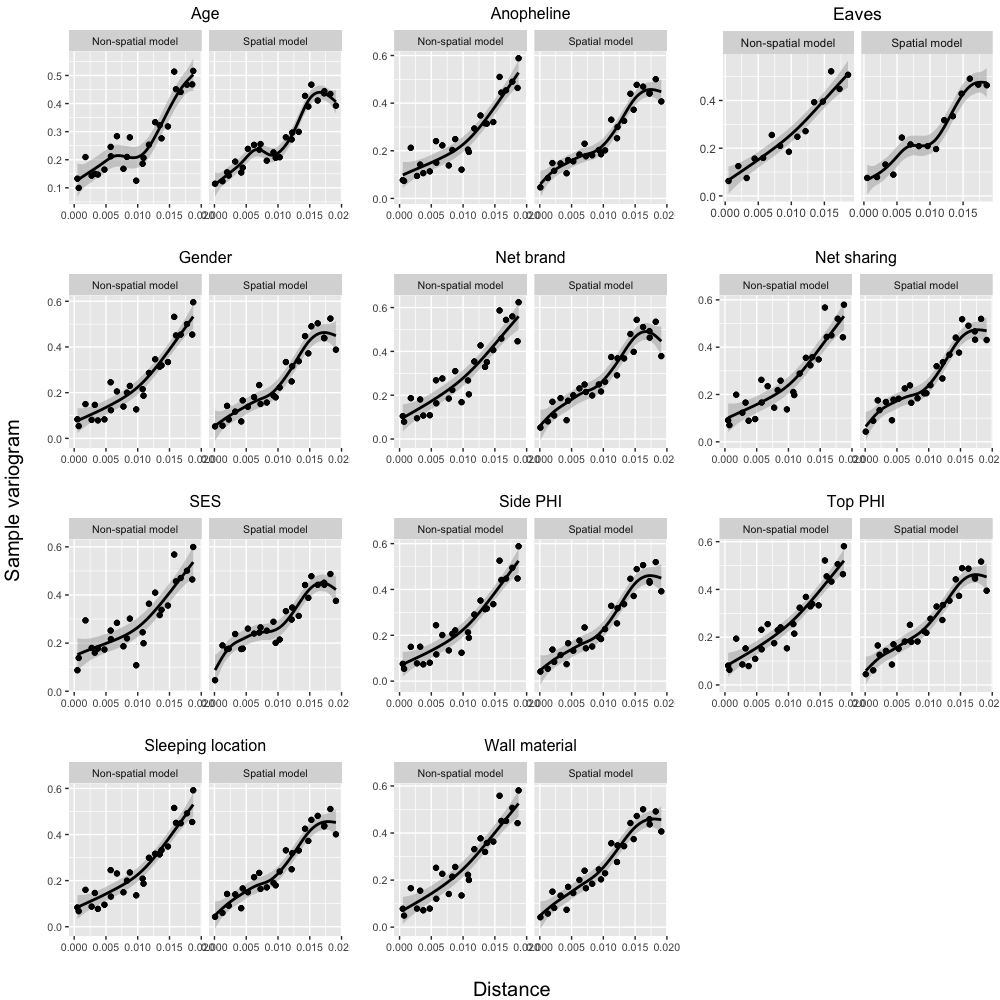

Supplement: Supplementary file 1 — Additional file 1: Fig. S1. Variograms to examine spatial dependency in the simple regression model for each explanatory variable. [file 12936_2020_3444_MOESM1_ESM.png]

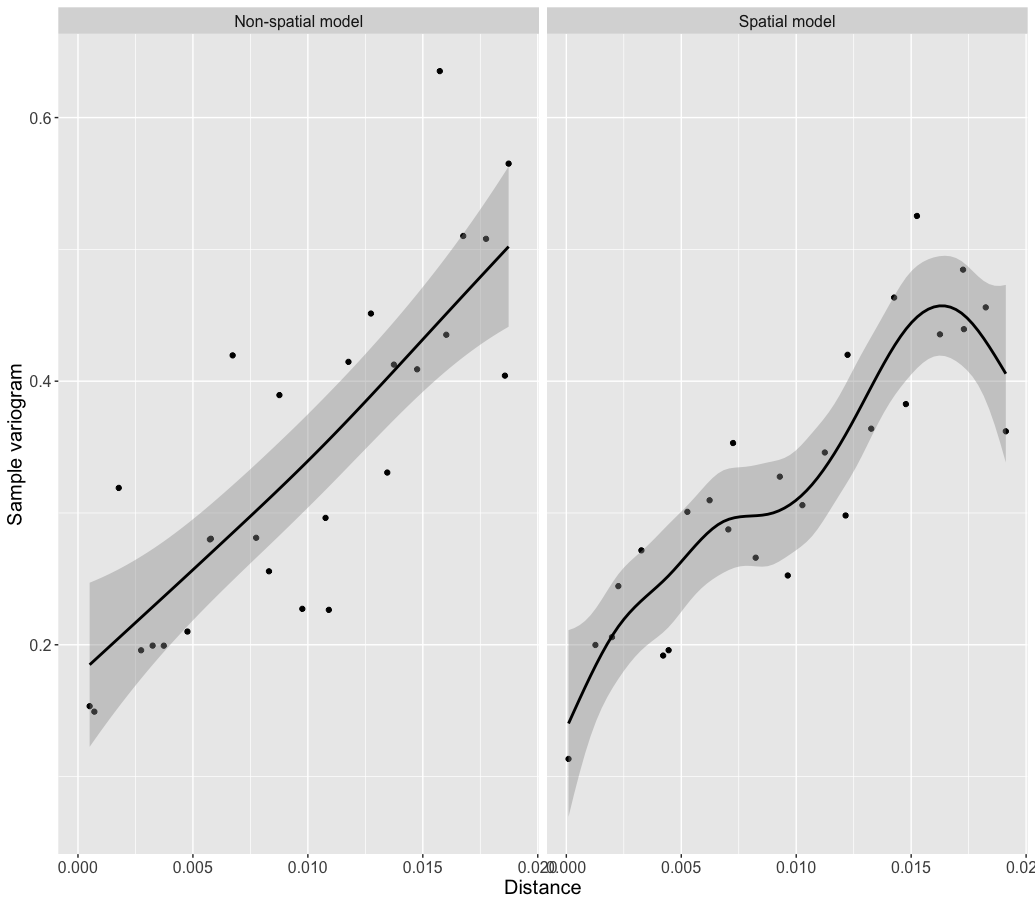

Supplement: Supplementary file 2 — Additional file 2: Fig. S2. Variograms to examine spatial dependency in the multiple regression model. [file 12936_2020_3444_MOESM2_ESM.png]

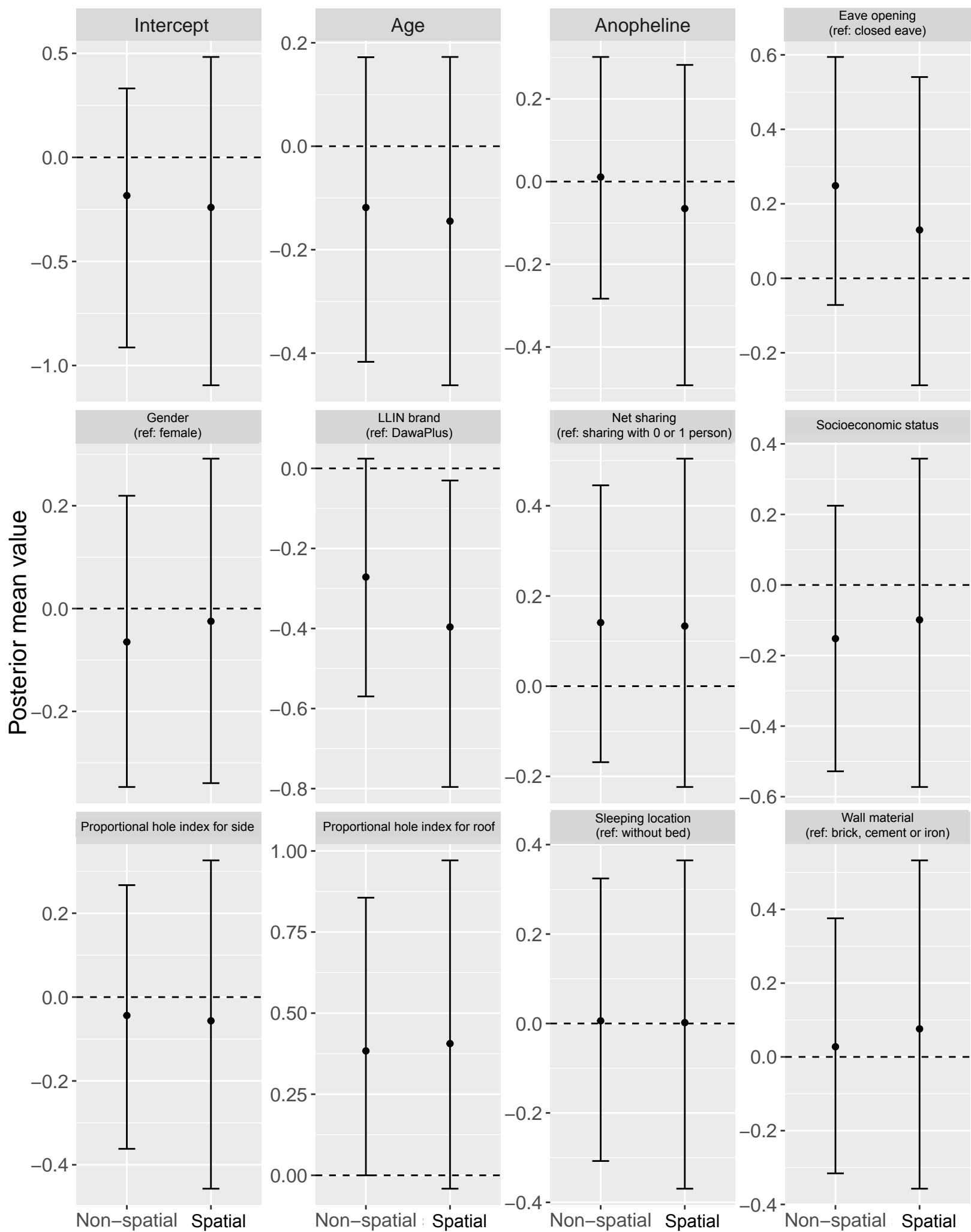

Supplement: Supplementary file 4 — Additional file 4: Fig. S4. The 95% credible intervals of each variable from non-spatial and spatial multiple regression models. [file 12936_2020_3444_MOESM4_ESM.pdf]
