# Supplementary material for: Protective effects of Olyset® Net on Plasmodium falciparum infection after three years of distribution in western Kenya
Source: Malar J. 2020 Oct 19;19:373. doi: 10.1186/s12936-020-03444-w (PMC7574443; doi:10.1186/s12936-020-03444-w)
Supplement: Supplementary file 3 — Additional file 3: Fig. S3. The 95% credible intervals of each variable from non-spatial and spatial bivariate regression models. [file 12936_2020_3444_MOESM3_ESM.pdf]

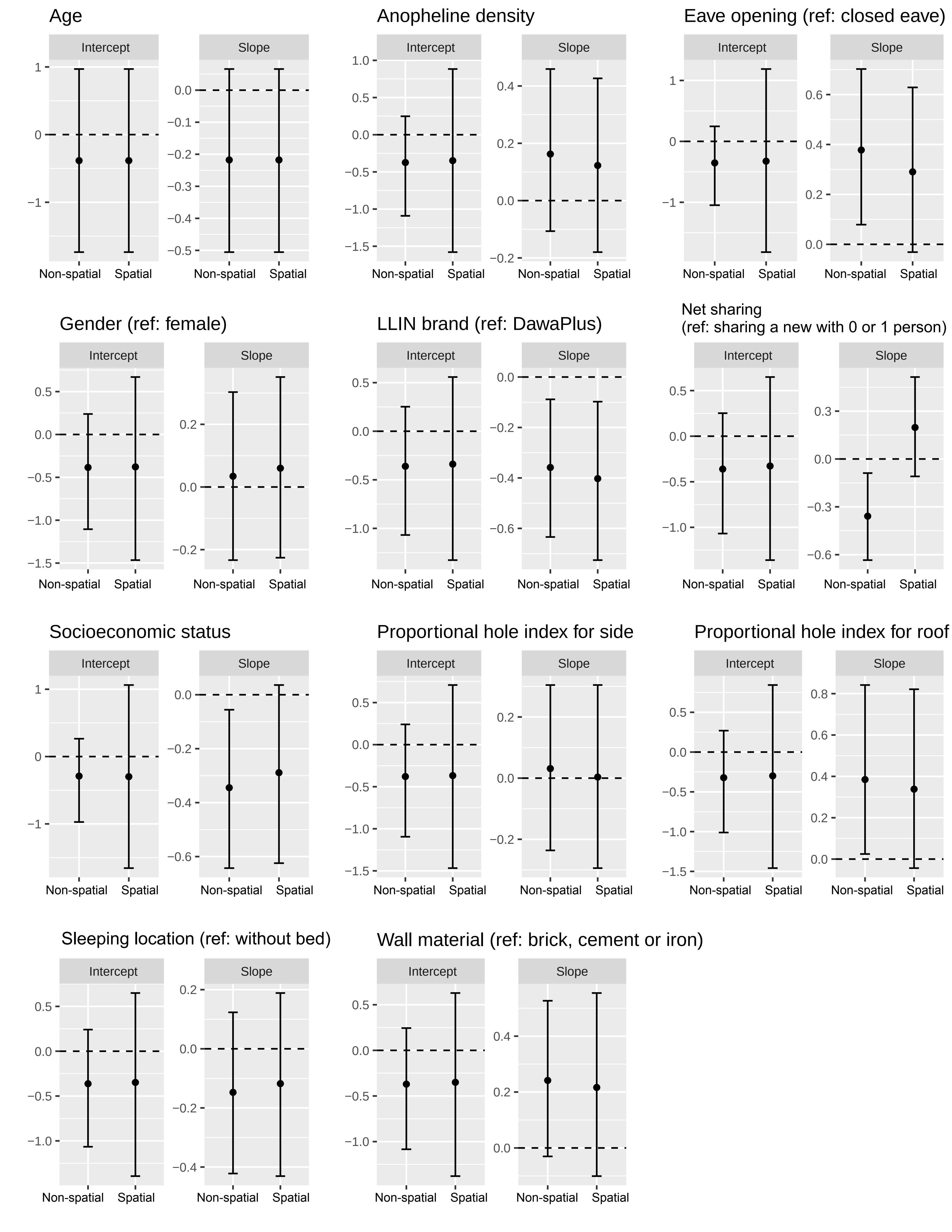

Age
Intercept
Slope
Non-spatial
Spatial
Anopheline density
Eave opening (ref: closed eave)
Gender (ref: female)
LLIN brand (ref: DawaPlus)
Net sharing
(ref: sharing a new with 0 or 1 person)
Socioeconomic status
Proportional hole index for side
Proportional hole index for roof
Sleeping location (ref: without bed)
Wall material (ref: brick, cement or iron)
